# The Role of Mobile Applications in Enhancing the Health-Related Quality of Life of Children with Cancer: A Systematic Review and Meta-Analysis

**DOI:** 10.3390/children12070927

**Published:** 2025-07-14

**Authors:** Ana González-Díaz, Bibiana Pérez-Ardanaz, Nora Suleiman-Martos, José L. Gómez-Urquiza, Cristina Canals Garzón, Juan Gómez-Salgado

**Affiliations:** 1 Faculty of Health Sciences, University of Granada, 51001 Ceuta, Spain; agonzalezd@ugr.es (A.G.-D.); bibinap@ugr.es (B.P.-A.); ccanals@ugr.es (C.C.G.); 2 Faculty of Health Sciences, University of Granada, 18071 Granada, Spain; norasm@ugr.es; 3 Department of Sociology, Social Work and Public Health, Faculty of Labour Sciences, University of Huelva, 21007 Huelva, Spain; salgado@uhu.es; 4 Safety and Health Postgraduate Programme, Universidad Espíritu Santo, Guayaquil 092301, Ecuador

**Keywords:** smartphone, mobile applications, child health, neoplasms, digital health, systematic review

## Abstract

**Background/Objectives**: Childhood cancer, although relatively rare, has a profound impact on the quality of life of affected children and their families. Technological advances have facilitated the development of mobile applications (apps) aimed at enhancing symptom monitoring and improving communication with healthcare teams. This systematic review aimed to analyse the effect of mobile applications on the health of children with cancer, with a specific focus on health-related quality of life (HRQoL). **Methods**: A systematic review was conducted in accordance with PRISMA 2020 guidelines. Searches were performed in PubMed (Medline), CINAHL, Cochrane and Scopus databases using MeSH terms such as Smartphone, Mobile Applications, Child Health, Neoplasms, and Digital Health, with no date restrictions, and including studies published in English, Spanish or Portuguese. We included original research studies that examined the use of mobile apps in paediatric oncology patients. The search was completed in January 2025. **Results**: Of the 324 records initially identified, 14 studies (mainly pilot studies, early-phase clinical trials, and observational designs) met the inclusion criteria. Interventions commonly focused on symptom tracking (pain, nausea, fatigue), promoting treatment adherence, and delivering educational content. Several studies reported high user acceptance and a potential positive impact on HRQoL, particularly when gamification strategies were incorporated to sustain children’s engagement. **Conclusions**: Despite the preliminary nature and small sample sizes of most studies, mobile applications appear to be effective in supporting symptom management, communication, and health education in paediatric oncology. Their use may contribute to improvements in HRQoL. Further high-quality research involving younger children and diverse socio-cultural contexts is required to confirm their effectiveness.

## 1. Introduction

Although rare, childhood cancer has a major impact on both survival and quality of life. Each year, between 360,000 and 400,000 new cases of childhood cancer are diagnosed, with leukemia representing approximately 25% of them [1,2]. Thanks to therapeutic advances, survival rates have improved significantly, now exceeding 85% in high-income countries [2]. Nevertheless, substantial disparities persist in middle- and low-income regions.

This increase in survival has highlighted the need to address not only the disease itself, but also the physical and psychological aspects that affect the quality of life of paediatric patients [3,4,5].

In particular, pain resulting from treatment—such as chemotherapy and surgery, or complications such as mucositis—is often more intense than that caused by the neoplasm itself [3]. This pain has additional adverse effects, such as sleep disturbances, fatigue, fear and anxiety, which significantly impair the health-related quality of life (HRQoL) of these children [4,5]. HRQoL is defined as how illness and its treatment affect a child’s physical, emotional, social, and overall well-being [2].

In this context, paediatric care requires a comprehensive approach that involves the child, their family and the healthcare team. This requires precise, dynamic communication strategies tailored to the patient’s age [6]. Traditionally, play and playful methods have been key tools for facilitating expression and learning in childhood [7]. However, in recent years, new digital strategies have gained prominence.

Among these new strategies, the use of mobile technologies as a complementary therapeutic resource in paediatric oncology stands out. Unlike traditional methods, these tools allow for more constant monitoring of the child’s condition and encourage the patient’s active participation in their own care process [8,9].

Electronic tools, such as mobile applications (apps), are currently being explored to improve symptom detection and recording, as well as to provide emotional and educational support. The use of smartphones and tablets is widespread, even among young children, who have access to their own devices from the age of 11 or share their parents’ devices [8,9]. These tools offer advantages such as real-time communication, immediate data collection, and the possibility of creating peer support networks [10].

In paediatric oncology, the use of apps has been explored for various purposes: pain measurement, symptom and side effect monitoring, promoting treatment adherence, and reinforcing disease education [11,12,13,14].

However, there is a wide variety of applications and uses for these tools. The tools used do not have a theoretical basis or a common objective, thus limiting their clinical application and usefulness. Furthermore, most studies focus on short-term results, leaving questions about their sustained impact on quality of life.

Though, despite their potential, evidence on their specific impact on the quality of life of children with cancer is still limited. This highlights the need for further research to evaluate their effectiveness and feasibility in this clinical context. In this regard, it is essential to systematise the current knowledge available on the use of these technologies to identify which components have proven to be effective, which gaps remain in the literature, and which criteria should be considered for their safe and ethical implementation in hospital settings.

Therefore, the following research question is posed: What is the effect of mobile app use on the health, and especially the quality of life, of children with cancer?

Consequently, the overall objective of this review is to analyse the impact of mobile applications on the health of children with cancer, including their potential contribution to improving communication, treatment adherence, and HRQoL.

## 2. Materials and Methods

A systematic review was conducted following the PRISMA 2020 guidelines (Preferred Reporting Items for Systematic Reviews and Meta-Analyses) (Page et al., 2021 [15]). The review was registered in OSF Registries.

### 2.1. Information Sources and Search Strategy

The PUBMED (Medline), CINHAL (Cumulative Index of Nursing and Allied Health Literature), SCOPUS, and Cochran databases were consulted. To construct the search equation, terms from the MESH (Medical Subject Headings) thesaurus were used and combined with Boolean operators. The final search was as follows: (((((((Neoplasms[MeSH Terms]) OR (oncology[Title/Abstract])) OR (Pediatric oncology[Title/Abstract])) OR (pediatric cancer[Title/Abstract])) OR (tumor[Title/Abstract])) OR (cancer[Title/Abstract])) AND ((((child health[MeSH Terms]) OR (children[MeSH Terms])) OR (child health[Title/Abstract])) OR (child*[Title/Abstract]))) AND ((((((((((Mobile Applications[MeSH Terms]) OR (Smartphone[MeSH Terms])) OR (Digital Health[MeSH Terms])) OR (Mobile Applications[Title/Abstract])) OR (Smartphone[Title/Abstract])) OR (Digital Health[Title/Abstract])) OR (Digital health interventions[Title/Abstract])) OR (mHealth app[Title/Abstract])) OR (Smartphone App[Title/Abstract])) OR (Mobile apps[Title/Abstract])). The search was conducted in January 2025 with no restrictions on year of publication. Articles in Spanish, English, and Portuguese were considered.

### 2.2. Eligibility Criteria

Inclusion criteria: Studies analysing the use of mobile applications or smartphones in paediatric cancer patients (under 18 years of age), original articles with a quantitative, qualitative or mixed design (clinical trials, observational studies, pilot studies, case–control studies, descriptive studies).

Exclusion criteria: Systematic reviews, meta-analyses or theoretical articles that did not provide original data on paediatric cancer samples, studies focused on the adult population without differentiating between results for children and adolescents, and documents that do not explicitly address cancer as the main pathology.

### 2.3. Study Selection Process

The articles were selected in several stages. First, duplicates were eliminated using the Zotero bibliographic manager (7.0.16), and then some of these studies were discarded by reading the title and abstract. After this, the selected primary studies were read in full and critically to confirm their final inclusion. Finally, those studies that met all the inclusion criteria were selected. The selection process was performed by two members of the research team (AGD and JLGU) independently to ensure consistent application of the eligibility criteria.

### 2.4. Data Extraction and Analysis

The information from the selected studies was collected in a table with the following variables: authors, year of publication, country, type of study, main objective, sample size and characteristics of the population, characteristics of the intervention (type of application, main functionalities), results, conclusions, and level of evidence. A descriptive analysis of the data obtained was used for the systematic review. For the meta-analysis, StatsDirect software (version 4.0.4) was used, calculating the prevalence of ease of use of mobile applications as the effect size, which shows how easy these technologies can be implemented. A fixed-effects meta-analysis was used, and heterogeneity was assessed using I2. Publication bias was assessed with the Egger test. This was the only performed meta-analysis because the studies did not have the necessary statistical data to be combined for other outcomes.

### 2.5. Methodological Quality Assessment

To assess the quality of the included studies, the Mixed Methods Appraisal Tool (MMAT) was used, applying the relevant items according to the design type of each article. Each study was included if the answer to three MMAT [16] questions was positive. In addition, the degree of recommendation and level of evidence were assigned using the Oxford Centre for Evidence-Based Medicine (OCEBM) scale, showing how strong is the evidence of each study.

The results of this assessment are presented in Appendix A, including the most relevant aspects regarding the quality and validity of the studies reviewed.

## 3. Results

### 3.1. Selection of Results

The initial search of the databases identified 446 documents. After removing 133 duplicate articles, 313 records remained.

**Figure 1:**
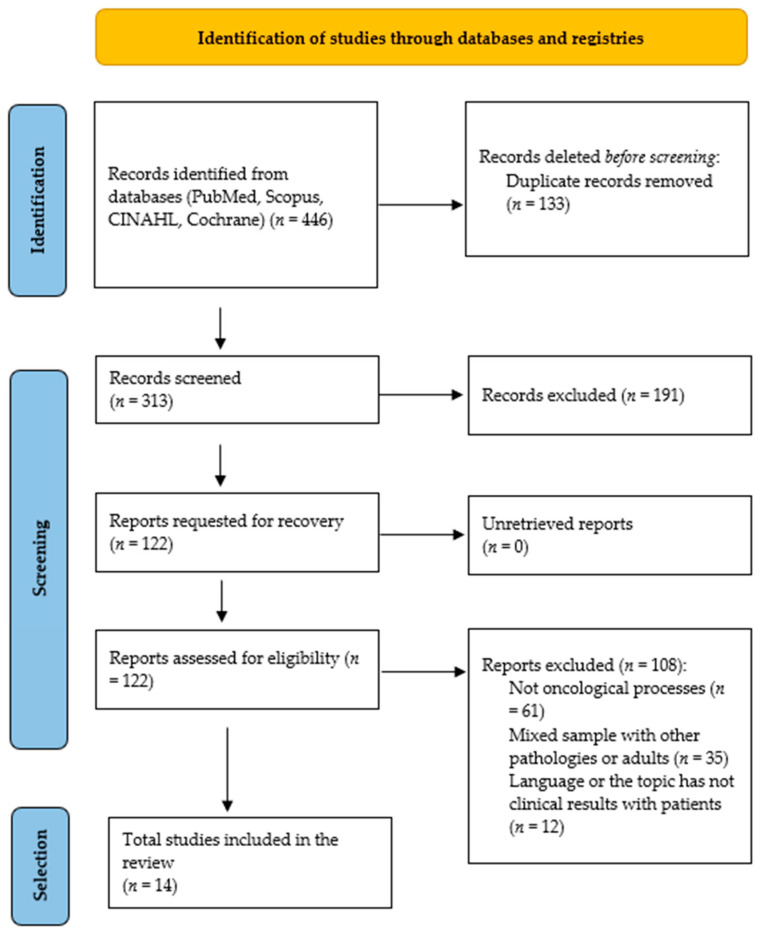
Flow diagram of study selection.

Subsequently, after reading the title and abstract, 191 documents that did not meet the inclusion criteria were eliminated. Of these, 122 studies were read in full. Finally, 108 were excluded because they did not meet the eligibility criteria, did not use mobile applications for oncological processes, did have a mixed sample that included adults or other pathologies, or if the language or the topic was on the development of an app, without clinical results/patients. As a result, 14 studies were included in the qualitative synthesis (Figure 1).

### 3.2. Characteristics of the Studies

Table 1 describes the main characteristics of the 14 studies included, indicating the author, year and country of publication, type of design, sample, objective, main intervention, results and conclusions, as well as the level of evidence and degree of recommendation.

Most of the studies analyse the usefulness of mobile applications (apps) focused on paediatric cancer patients, with the aim of recording or assessing symptoms (e.g., pain, nausea, fatigue, vomiting, mucositis); improving communication between patients, families, and healthcare professionals; and providing support or education to reduce anxiety and improve health-related quality of life (HRQoL).

In terms of geographical distribution, most studies were conducted in Canada and the United States, with three studies in Europe (United Kingdom, Denmark, and Germany) also noteworthy. The samples ranged from 3 to 94 paediatric participants, mainly over the age of 8, with an age limit of 18 years. Several studies were presented as pilot studies, case studies, or preliminary clinical trials, highlighting the need for further large-scale research.

## 4. Main Findings

To summarise the results, the studies have been grouped according to the main objective of the mobile application:

### 4.1. Symptom-Specific Monitoring Through Mobile Applications

Aldiss et al., 2011 [17], with ASyMS and Baggot et al., 2021 with eDiary [18] and Simon et al., 2021 and 2024 with Klick Pain Monitor [25,30] are examples of apps designed to collect information and manage the main symptoms, such as pain, nausea and sleep quality. Its use proved viable and was well received by patients, who described the app as ‘easy to use’, “rewarding” and ‘interesting’ despite some limitations in terms of scalability. Adherence exceeded 85% in all app examples. All these app examples used an automatic alarm system based on the severity of the symptom.

Tomlinson et al., 2014, with eCHIMES, [20] focused specifically on the assessment of mucositis, while the PicPer app [29] created apps to measure nausea and vomiting in real time, with a high response rate and good acceptance among children, enabling better communication and reducing discomfort by 30%.

### 4.2. Mobile Apps for Pain Assessment and Management

Some studies, e.g., Stinson et al., 2013 and 2015, focused on the Pain Squad app or its improved version Pain Squad+ as in Jibb et al., 2017 and 2018 [19,21,22,23]. These apps integrate gamification and positive reinforcement elements to assess and manage pain. They were found to promote self-management, increase adherence to recording, and can improve quality of life by reducing the impact of pain and associated distress. They have been shown to have high validity and reliability for assessing pain and sensitivity to post-surgical changes [24] based on qualitative results, as reported by PainBudi, as in Jibb et al. 2018, [23] where after interviewing 20 children, they obtained responses such as “The app helped me feel like I was in control when I was in pain”, “I liked earning rewards, it made me want to keep using it”, or, from Stinson 2013, [19] who obtained similar results: “Gamification helped me want to keep doing it every day”, or “playing detective made it fun, not like a chore”.

In the Pain Buddy app belonging to Hunter et al., 2020 [24], the frequency of moderate-to-severe pain decreased in the intervention group after using the app. Likewise, in Simon et al., 2021 [25], with the KLIK app, patients were able to record their pain from home with automated clinical alerts. The app demonstrated good levels of professional adherence and a rapid response, resulting in faster pain management.

### 4.3. Meta-Analysis on the Ease of Use of Applications

**Figure 2:**
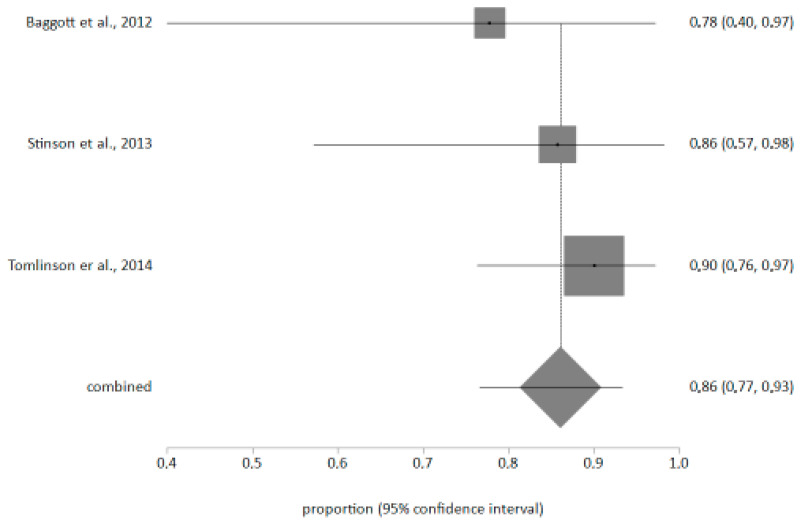
Meta-analysis of the prevalence of “very easy use” of the mobile app [18,19,20].

The ease of use of mobile applications as perceived by children with cancer was assessed. There were n = 3 studies that included the statistical information necessary to calculate the effect size. With n = 63, an effect size of 86% (CI 77–93%) was obtained, indicating that the mobile application was very easy to use (Figure 2). The I2 value was 0%. [19,23,26], and the use of Pain Squad and iBounce led to high acceptance and usability among the paediatric population. The Egger test did not show publication bias (*p* > 0.1)

### 4.4. Level of Evidence and Methodological Quality

Most of the studies are pilot studies, preliminary clinical trials, or descriptive observational studies, which is reflected in their levels of evidence (B/2b) according to the Oxford scale, with two clinical trials reflecting level (A/1b). In all cases, a high level of methodological quality was observed according to the Mixed Methods Appraisal Tool (MMAT), as detailed in Annex I. Among the main levels of evidence, the following stand out:

The studies with the highest level of evidence were 1b and recommendation A, those carried out by [23,24]. Both studies demonstrated a significant reduction in pain in children with cancer. Use of the Pain Buddy app reported a value of 0 episodes of moderate-to-severe pain at the end of follow-up, while the control group maintained these episodes persistently (*p* = 0.007) [24]. The use of KLIK by Simon et al. [30] reduced clinically significant pain (>4 on the NRS-11 scale) with an odds ratio of 0.38 (95% CI: 0.198–0.734) and a reduction in pain severity with a β coefficient = −0.27 (95% CI: −0.407 to −0.142); in addition, parents showed less emotional distress (β = −0.84, 95% CI: −1.61 to −0.03).

The studies proposed by [23,29] with a level of evidence of B/2b and B/3b, respectively, were based on qualitative or descriptive designs without a control group or robust inferential analysis, analysing 8 and 14 children, focusing on subjective experience and pain management without conclusive quantitative data.

The main limitations regarding the level of evidence of the other studies were small sample size, absence of a control group in some studies, lack of long-term follow-up, and heterogeneity in the variables analysed. Nevertheless, the results proposed by Aldiss., 2011 [17], Baggott et al., 2012 [18], and Simon et al., 2021, 2024 [25,30] should not be overlooked, as studies with a C/4 level have offered significant results on the management of symptoms such as pain and nausea, as well as sleep quality, showing high patient acceptance, with adherence above 85%, Moreover, the studies of Tomlinson., 2014 [20] and Esplana, 2023 [29] demonstrated improved communication between children, caregivers, and professionals, as well as a 30% reduction in discomfort. Finally, Hunter et al., 2020 [24] demonstrated a positive impact on the reduction in moderate-to-severe pain and faster care thanks to automatic alert systems.

## 5. Discussion

The objective of this systematic review was to analyse the effect of mobile applications (apps) on the health of children with cancer, especially in relation to health-related quality of life (HRQoL). The results indicate that using apps designed to record symptoms (pain, nausea, fatigue, vomiting, mucositis) promotes therapeutic adherence, or it provides education and support children.

Firstly, apps designed to record and monitor pain, such as Pain Squad or Pain Squad+, are particularly useful [19,21,22,23], as they incorporate gamification elements and virtual rewards. Their ability to promote self-management and adherence to daily pain assessment suggests a promising approach to improving the management of the most prevalent symptom in paediatric oncology. Similarly, the incorporation of electronic diaries (eDiary) or specific applications for mucositis (eChIMES) and nausea (Pediatric Nausea Assessment Tool) has demonstrated feasibility and acceptance, facilitating early detection of complications by healthcare professionals [18,20,27]. This coincides with other studies that point to patients’ preference for electronic media over traditional paper methods, as they are more dynamic and interactive [28,29].

Another relevant finding is improved adherence to medical follow-up through appointment reminders or physical activity monitoring in cancer survivors [21,24]. These interventions encourage the active participation of children and adolescents and their caregivers, promoting healthy habits and better disease control. Although several studies show encouraging results, many of them are pilot studies or have small sample sizes, which limits the extrapolation of their findings.

In relation to HRQoL, although most research focuses on measuring specific symptoms (pain, nausea) or aspects of adherence, the authors suggest that symptom reduction, as well as the sense of control and autonomy provided by these applications, could have a positive impact on HRQoL [4,5,23,25]. In this regard, having more fluid communication tools, alarm systems for early detection of complications, and virtual spaces for mutual support can help reduce anxiety and fear, factors that often accompany cancer treatment in children [3,6,31].

Some qualitative studies have demonstrated the added value of mobile apps in addressing pain and symptoms, not only because of their functionality but also because children reported feeling more autonomous and empowered in managing their pain, expressing phrases such as “it helps me feel in control when I’m in pain” [23]. Adding gamification elements improved adherence through dynamic and playful rewards [19,23]. Similarly, ease of use, user-friendly design, and perceived usefulness were highlighted as positive attributes by participants, who described the apps as “easy to use, educational and interesting” [18].

However, barriers to everyday implementation were also identified: adolescents reported forgetting to use the apps in school settings or needing more adaptable reminders [22,23]. Similarly, although more exploratory, some studies suggest that these tools also provide a form of emotional support by allowing children to express their discomfort and feel accompanied in real-time [24]. These findings underscore the importance of considering not only the clinical efficacy of digital interventions but also their experiential and subjective dimension in the paediatric oncology context.

According to the meta-analysis, it was observed that the prevalence of satisfaction among children using mobile applications to record symptoms is high [18,19,20]. This may be due to the high use of technology among the current paediatric population [32].

Several studies have reported results similar to those obtained in our research, demonstrating that mobile applications are useful tools for managing symptoms in childhood cancer [12,13,33]. However, few applications have been designed specifically for the entertainment of children during treatment. Despite growing interest in this area, no studies have been found that have conducted meta-analyses on the use of these technologies. This reflects a gap in the literature that deserves further exploration.

Despite these promising results, some limitations should be considered. First, many studies are pilot studies with small samples or no control group. Second, there is a lack of research with younger populations (under 8 years of age), probably due to the difficulties involved in getting these children to actively participate in daily records and the need to adapt the content to their stage of development. Furthermore, the studies included focus mainly on developed countries; therefore, the findings may not be directly transferable to contexts with fewer technological resources. Finally, the heterogeneity of assessment methods (pain scales, symptom definitions, quality of life instruments) makes direct comparison between studies difficult. Finally, the meta-analysis only has three studies, and most of the studies do not report important statistical data about main clinical outcomes to perform a better meta-analysis.

In summary, mobile applications aimed at children with cancer have proven to be viable tools for symptom control, therapeutic adherence, health education, and anxiety reduction, potentially contributing to improved quality of life. However, larger-scale clinical trials with long-term follow-up are needed to more robustly analyse the efficacy and real impact of these interventions. Future research could also focus on developing apps tailored to younger age groups or with multimodal features that include psychological support, caregiver education, and even virtual or augmented reality environments.

Future research should be more experimental and show means and standard deviation postintervention. Moreover, larger samples and robust methodological designs, including long-term comparability and younger age groups, are needed to generalise the findings and further explore the specific impact of each type of application. It will also be important to explore settings with more limited technological resources and adapt interventions to different sociocultural contexts. In this way, evidence on the effectiveness of mobile apps as part of a comprehensive and multidisciplinary approach that promotes the quality of life of children with cancer and their families can be strengthened.

## 6. Conclusions

The use of mobile applications in paediatric oncology is emerging as a promising strategy for improving symptom recording and management (especially pain, nausea, and fatigue), as well as promoting treatment adherence and strengthening communication between children, their families, and healthcare teams. Although most of the studies included are pilot studies or have small samples, the results indicate that these tools are generally accepted and well regarded by children and their carers, helping to alleviate symptoms and increase the feeling of control over the disease.

Gamification and real-time feedback are proposed as key elements in motivating active participation and sustaining continued use of the apps, which results in better symptom reporting and, potentially, higher health-related quality of life (HRQoL). Benefits have also been identified in treatment adherence and disease education, which may translate into a reduction in the distress and anxiety typical of the cancer process.

## Figures and Tables

**Table 1:** Characteristics of the included studies (n = 14).

Author, Year of Publication and Country	Type of Study	Sample and Objective	Intervention	Result and Conclusions	GR/EV
Aldiss et al., 2011 [17] United Kingdom	Case–control study	To develop an alarm system based on the severity of symptoms in paediatric patients undergoing Advanced Symptom Management System (ASyMS). N = 3	The study had three phases, a first phase where they reflected on which symptoms were important to be aware of, a second phase where they reflected on their perceptions of the ASyMS system and a third phase where they designed an alarm system based on the severity of the symptoms.	The small number of participants did not allow for a meaningful analysis of the results, concluding that the self-care advice provided by the app was good and simple, but the alarm system that was attempted to be developed was not effective, thus stating the need for studies that allow for large-scale evaluations.	C/4
Baggott et al., 2012 [18] EE.UU	Case–control study	To find out the usefulness of an electronic diary that assessed daily pain, nausea, vomiting, fatigue and sleep quality, designed for cancer patients (eDiary) through a mobile application. N = 11	The adherence of the young people exceeded the expectations of the technical difficulties reported.	The usefulness of the diary for patients has been shown to improve the quality of care. It has been shown to improve patient management and the symptomatic relationship with cancer.	C/4
Stinson et al., 2013 [19] Canada	Prospective descriptive study	Test the construct validity, reliability and feasibility of the Pain Squad application. N = 92 N = 14	Two studies, the first one to assess construct validity and reliability of the application, had patients record their pain level twice a day. In the second, they recorded it 1 week before and 2 weeks after a surgical intervention.	The combined results of both studies support the app’s construct validity and reliability, demonstrating that it has good internal consistency. Furthermore, it was demonstrated that an electronic diary is advantageous for exploring pain levels in pediatric patients.	C/4
Tomlinson et al., 2014 [20] Canadá	Descriptive clinical trial	To determine the ease or difficulty of using the application (eChIMES) to achieve better development and greater effectiveness of the usage options. N = 40	Two phases were carried out: the first for the development and improvement of eChIMES (n = 10), and then to evaluate its ease of use, understandability, and suitability. Finally, the results were statistically analyzed using a descriptive analysis.	The results obtained were 100% easy or very easy to use, making it a good way to measure mucositis in children. It was considered innovative, a great idea, and easier than writing down one’s own symptoms or experiences. It was, therefore, concluded that eCHIMES was an easy-to-use and understandable application, but that it would be interesting to compare electronic versions with paper versions.	C/4
Stinson et al., 2015 [21] Canada	Prospective descriptive study	To design and develop a gamified mobile app, called Pain Squad, that allows patients to assess their pain and provide an immediate reward system. N = 47	Pain management drastically affected the social lives of participants, creating a disconnect between the healthcare system and the participants’ skills.	Participants with poor pain management demonstrated poor social role functioning, generating anxiety and stress in the parental relationship. The app demonstrated a development of the patient’s pain management skills. By improving social support with parents and improving pain education, it is concluded that the gamification of mobile healthcare can enable these patients to make better decisions about their treatment.	B/2b
Jibb et al., 2017 [22] Canada	Pilot study	To assess the implementation of an app (Pain Squad+) to inform a future randomized controlled trial (RCT) N = 38	Use of Pain Squad+, a 22-item questionnaire that assesses pain (duration and location), causes, and treatment strategies.	This study demonstrated that the application improves pain intensity, responding positively to the hypothesis raised, suggesting that this application can be implemented for a future RCT intervention.	B/2b
Jibb et al., 2018 [23] Canada	Qualitative pilot study	To understand the perception of adolescents with cancer and to understand their adaptability to the application, improving the protocol. N = 40	Participants agreed that the application would improve the protocol in a study with larger participants.	Although the results were approved by the participants, improvements in the intervention emerged in improving the quality of life of adolescents with cancer. Active participation in answering the questionnaire on symptom management and self-care improved quality of life, allowing them to seek help when necessary. Although the results were approved by the participants, improvements in the intervention emerged and there was an improvement in the quality of life of adolescents with cancer.	B/3b
Hunter et al., 2020 [24] USA	Qualitative pilot study	To evaluate the preliminary efficacy of Pain Buddy, an app for managing cancer-related pain in children. N = 48	Pain Buddy allows for remote symptom monitoring and offers pain management coaching. A 60-day randomized controlled trial was conducted, in which children reported their pain through a digital diary.	Both groups showed a reduction in pain severity, but the intervention group reported fewer episodes of moderate to severe pain. It is considered an innovative and interactive application that can, therefore, help reduce both the severity and frequency of pain.	A/1b
Simon et al., 2021 [25] Netherlands	Feasibility study	To evaluate adherence, feasibility, and barriers and facilitators to the implementation of an app designed to monitor and track pain in children with cancer at home. N = 27	The KLIK Pain Monitor app was used for three weeks. Children aged 8–18 years or their parents (for children aged 0–7 years) assessed pain twice daily using an 11-point numeric rating scale (NRS-11). Clinically significant pain scores (≥4) were monitored by healthcare professionals from the hospital’s Pediatric Pain Service within 120 min (scores of 4–6) or 30 min (scores of 7–10).	Sixty-three percent of families (17 of 27) used the app daily during the three weeks, and 18.5% (5 of 27) reported pain scores twice daily during that period. 44.4% of children (12 of 27) reported at least one clinically significant pain score. In 70% of cases (14 of 20) with clinically significant pain scores, healthcare professionals followed up within the established timeframe. Most of the app’s features were positively evaluated by at least 70% of families and healthcare professionals, and non-feasible aspects were resolved.	C/4
Ha et al., 2022 [26] Australia	Pilot study	To evaluate the feasibility and acceptability of the iBounce digital intervention, designed to educate and motivate survivors to participate in physical activity. N = 30	A digital educational program with 10 modules, goal setting, and home-based physical activities monitored by an activity tracker. Participants were instructed to complete modules and log their physical activity.	High module completion rate, with the app (iBounce) considered feasible and acceptable. Technical difficulties were encountered with the activity trackers, but it is considered a useful model for delivering physical activity education and promotion to cancer survivors.	B/3b
Linder et al., 2022 [27] EE.UU	Estudio descriptivo	To evaluate the feasibility and usefulness of a game-based application for children with cancer to report their symptoms and daily experiences during treatment. N = 19	The mobile application “Color Me Healthy” was used, designed to allow children to self-report their symptoms and daily experiences during cancer treatment.	The 19 children completed 107 days of app use. All reported symptoms at least once, and 14 reported at least one day with a moderate or greater symptom severity. The most frequent symptoms were pain (100% of children, 55.2% of days), fatigue (78.9% of children, 47.1% of days), and nausea (57.9% of children, 34.5% of days). Furthermore, reported daily experiences reflected children’s participation in usual childhood activities while also describing life with cancer, including symptoms.	C/4
Mehdizadeh et al., 2023 [28] Irán	Pilot study	To evaluate the usability of the Can-SelfMan app, designed to improve communication between caregivers and healthcare providers. N = 44	The app (Can-SelfMan) provides access to cancer information and symptom-tracking tools. It was used for three weeks, recording symptoms and asking questions answered by oncologists.	It proved to be a promising tool for improving care management, but there was a desire to improve interaction, so the application needs to be improved.	B/3b
Esplana et al., 2023 [29] Sweden	Pilot study	To evaluate children’s acceptability and experience of using the PicPecc app to manage nausea N = 8	Use of the PicPecc app in children with cancer during an observation period, allowing self-reporting of nausea with an interactive digital interface.	The app improved children’s communication about their condition and allowed them to be more involved in their care. There was a 30% decrease in reported nausea compared to baseline, with user satisfaction at 85%.	B/3b
Simon et al., 2024 [30] Netherlands	Non-randomized clinical trial	To evaluate whether the use of a pain monitoring app reduces clinically significant pain in children with cancer at home. N = 94	Use of the KLIK Pain Monitor app, which allows patients and their families to record moments of significant pain and receive real-time feedback from healthcare professionals.	The group using the app reported significantly less clinically significant pain and lower pain severity compared to the control group. Use of the app resulted in less clinically significant pain at home. Further research into the specific mechanisms of the app is recommended.	A/1b

Notes: GR = grade of recommendation; EV = level of evidence.

## Data Availability

Not applicable.

## Abbreviations

HRQoL	health-related quality of life

## Appendix A

**Study**	**Item 1**	**Item 2**	**Item 3**	**Item 4**	**Item 5**
[17]	Y	Y	Y	Y	Y
[18]	Y	Y	Y	Y	Y
[19]	Y	Y	Y	Y	Y
[20]	Y	Y	Y	Y	Y
[34]	Y	Y	Y	Y	Y
[21]	Y	Y	Y	Y	Y
[22]	Y	Y	Y	Y	Y
[35]	Y	Y	Y	Y	Y
[23]	Y	Y	Y	Y	Y
[36]	Y	Y	Y	Y	Y
[37]	Y	Y	Y	Y	Y
